# Rapid Evolution of Autosomal Binding Sites of the Dosage Compensation Complex in *Drosophila melanogaster* and Its Association With Transcription Divergence

**DOI:** 10.3389/fgene.2021.675027

**Published:** 2021-06-14

**Authors:** Aimei Dai, Yushuai Wang, Anthony Greenberg, Zhongqi Liufu, Tian Tang

**Affiliations:** ^1^State Key Laboratory of Biocontrol and Guangdong Key Laboratory of Plant Resources, School of Life Sciences, Sun Yat-sen University, Guangzhou, China; ^2^Bayesic Research, Ithaca, NY, United States

**Keywords:** dosage compensation complex, positive selection, pleiotropy, transposable elements, *Drosophila*

## Abstract

How pleiotropy influences evolution of protein sequence remains unclear. The male-specific lethal (MSL) complex in *Drosophila* mediates dosage compensation by 2-fold upregulation of the X chromosome in males. Nevertheless, several MSL proteins also bind autosomes and likely perform functions not related to dosage compensation. Here, we study the evolution of MOF, MSL1, and MSL2 biding sites in *Drosophila melanogaster* and its close relative *Drosophila simulans*. We found pervasive expansion of the MSL binding sites in *D. melanogaster*, particularly on autosomes. The majority of these newly-bound regions are unlikely to function in dosage compensation and associated with an increase in expression divergence between *D. melanogaster* and *D. simulans*. While dosage-compensation related sites show clear signatures of adaptive evolution, these signatures are even more marked among autosomal regions. Our study points to an intriguing avenue of investigation of pleiotropy as a mechanism promoting rapid protein sequence evolution.

## Introduction

The rate and mechanism of protein sequence evolution are central questions in evolutionary biology. Empirical data have shown that essential genes do not evolve more slowly than non-essential genes (Greenberg et al., 2008; Zhang and Yang, 2015). This supports the view that the rate of protein sequence evolution depends primarily on the level of functional constraint (Zhang and Yang, 2015; Wollenberg Valero, 2020), rather than on the level of functional importance (Karp et al., 2008). Among many correlates of protein evolutionary rate (Zhang and Yang, 2015), pleiotropy has long been recognized as an important mechanism constraining protein evolution (He and Zhang, 2006). Amino acid sequences of highly pleiotropic (i.e., influencing many phenotypes) genes evolve relatively slowly (He and Zhang, 2006), due to the potential deleterious effects of mutations at these loci on additional traits. However, synergistic effects of some genes on multiple phenotypes can override the costs of complexity (McGee et al., 2016) and facilitate rapid adaptation (Archambeault et al., 2020). To better understand how pleiotropy shapes adaptation driven by rapidly evolving proteins, it is important to examine a variety of cases in depth. Sex chromosome evolution and genetic conflicts are fertile grounds to find examples of fast adaptation. Dosage compensation, the process whereby expression of sex-linked genes is equalized in both sexes, brings together evolutionarily labile sex determination and constrained fundamental transcriptional regulation. It is thus a promising place to look for fast-evolving pleiotropic genes.

The Male-Specific Lethal (MSL) or Dosage Compensation Complex (DCC) in *Drosophila* upregulates transcription from male X chromosomes to equal the two X chromosomes in females. MSL-DCC in this study is used specifically to refer to the MSL complex that functions in dosage compensation. It is composed of five proteins [MSL1, MSL2, MSL3, MOF (males absent on the first), and MLE (maleless)] and two long non-coding RNAs (*roX1* and *roX2*). MSL1 and MSL2 are required for scaffolding the MSL complex and targeting it to the X chromosome (Lyman et al., 1997; Scott et al., 2000). This targeting enables MOF, a histone H4 lysine 16-specific acetyltransferase, to induce transcriptional up-regulation through histone modification (Larschan et al., 2011). Two models can explain how the MSL complex achieves X dosage compensation (Lee and Oliver, 2018). In one, the MSL complex directly boosts gene expression primarily via enhanced elongation of transcription (Larschan et al., 2011). Alternatively, MSL proteins indirectly mediate dosage compensation by triggering an inverse dosage effect through MOF sequestration to counteract the potential over-expression of X-linked genes (Sun et al., 2013a,b). In both models, the MSL complex binds to the male X at high-affinity (HAS) or chromosome entry sites (CES) and then spreads to the entire chromosome (Alekseyenko et al., 2008; Straub et al., 2008). Loss-of-function mutations in each of the five MSL protein-coding genes result in male-specific lethality (Belote and Lucchesi, 1980; Skripsky and Lucchesi, 1982; Hilfiker et al., 1997). Given their essential role in male function, MSL proteins are expected to be highly constrained and under purifying selection.

Despite their essential functions, all five genes encoding the MSL proteins evolved adaptively on the *Drosophila melanogaster* branch (Levine et al., 2007; Rodriguez et al., 2007). It is unclear whether selection acts on the dosage compensation function itself or on an independent function carried out by either an MSL protein or an interacting gene product (Levine et al., 2007). While its role in dosage compensation is well-documented, there is reason to believe that the MSL complex has additional functions, as suggested, for example, by the expression of all MSL protein subunits except MSL2 in females (Prestel et al., 2010). Interestingly, there are three modes of MSL protein binding depending on chromatin context (Straub et al., 2013). First, MSL2 and MLE establish the primary contact that defines high-affinity sites for the MSL-DCC whereas MSL1 and MOF associate more indirectly (Straub et al., 2013). Second, MSL3 mediates the association of the MSL-DCC with actively transcribed gene bodies in an HAS-dependent manner (Straub et al., 2013). Third, MOF and MSL1 bind to active promoter regions across the genome with no chromosomal preference (Straub et al., 2013). The MSL1-MOF binding at promoters is independent of MSL2, clearly indicating their function outside of dosage compensation. In addition, MOF is associated with autosomes as part of the non-specific lethal (NSL) complex (Cai et al., 2010; Raja et al., 2010), binds to many housekeeping genes in both sexes (Feller et al., 2012; Lam et al., 2012), and plays a role in transcriptional noise reduction (Lee et al., 2018).

These distinct, although perhaps mechanistically linked, MSL complex functions provide us with an opportunity to study how different selection pressures shape MSL protein evolution. This should help us understand how pleiotropy influences adaptive protein sequence evolution. At the molecular level, pleiotropy may represent the necessity for a protein to bind to multiple interacting partners. Binding sites of a protein can thus shed light on the impact of pleiotropy on its sequence and functional evolution. While evolution of the MSL complex binding sites has been documented in *Drosophila* species (Rodriguez et al., 2007; Bachtrog, 2008; Alekseyenko et al., 2013; Ellison and Bachtrog, 2013, 2019; Quinn et al., 2016), little is known about the effect of pleiotropy on intensity of positive selection. Using MOF, MSL1, and MSL2 ChIP-seq data (Figueiredo et al., 2014; Chlamydas et al., 2016), we examined MSL-binding site evolution between *D. melanogaster* and *D. simulans* by classifying these sites as those involved in dosage compensation and those performing unrelated functions (hereafter referred to as DC and non-DC sites, respectively). We show that while both groups of sites have evolved rapidly on the *D. melanogaster* branch, non-DC sites harbor stronger signatures of positive selection. A substantial fraction of non-DC sites in *D. melanogaster* overlaps *cis*-regulatory elements and/or transposable elements (TEs), and is associated with increased expression divergence between *D. melanogaster* and *D. simulans*. These findings support the idea of co-evolution of DNA-protein interactions of the *Drosophila* MSL complex and suggest that selection for gene expression regulation, independent of dosage compensation, has contributed to adaptive evolution of MSL proteins in *D. melanogaster*.

## Materials and Methods

### Calling Binding Peaks of MSL Proteins

MOF, MSL1, and MSL2 ChIP-seq data collected from male third instar larval salivary glands of *D. melanogaster* and *D. simulans* were retrieved from Figueiredo et al. (2014) and Chlamydas et al. (2016) (Supplementary Table 1). Raw ChIP-seq reads were first trimmed for quality using Trimmomatic (version 0.36) (Bolger et al., 2014) with parameter “LEADING:3 TRAILING:3 SLIDINGWINDOW:4:15 MINLEN:25,” and then mapped to whole genomes of *D. melanogaster* (Ensembl BDGP6) and *D. simulans* (FlyBase r2.02) using bowtie (version 1.1.2, -a -m 1) (Langmead et al., 2009). Binding peaks were called using macs2 (version 2.1.1.20160309, callpeak -B –nomodel –SPMR -g dm) (Zhang et al., 2008). Each peak was considered as a binding site as there is only one sample for ChIP-seq of each protein in each species. Cross-correlation analysis was performed using version 1.15.2 of SPP (Kharchenko et al., 2008) with the default parameter of “-s= -100:5:600.”

### Comparative Genomic Analysis of MSL Binding Sites Between *D. melanogaster* and *D. simulans*

We estimated the gain or loss of the bound regions of each MSL protein following Bradley et al. (2010) with some modifications. Briefly, we calculated binding signals as the linear scaled fold enrichments by macs2 (version 2.1.1.20160309, bdgcmp -c treat_pileup.bdg -t control_lambda.bdg -m FE) (Zhang et al., 2008). The output files of “macs2 callpeak -B –nomodel –SPMR -g dm” were used as the treat and control .bdg files. For each bound region in each species, we searched the highest binding signal in the region in the source species and in the orthologous region of the other species. We extended the orthologous region by its half length on each side to capture the highest binding signal in the other species. Peaks were called as absent if the binding signal was reduced 10-fold or more in its ortholog.

Furthermore, binding sites in one species were mapped onto the orthologous regions of the other. Binding sites were considered conserved if at least half of its binding region in one species overlapped the orthologous region that was also bound by the same MSL protein in the other species (Sundaram et al., 2014). Otherwise, the binding sites were considered unconserved. The reciprocal best chains of *D. simulans* to *D. melanogaster* (http://hgdownload.cse.ucsc.edu/goldenpath/dm3/vsDroSim2/reciprocalBest/) and bnMapper (https://github.com/bxlab/bx-python/blob/main/scripts/bnMapper.py, Denas et al., 2015) were used to do the one-to-one ortholog mapping.

### Determining DC and Non-DC Sites

**Figure 1:**
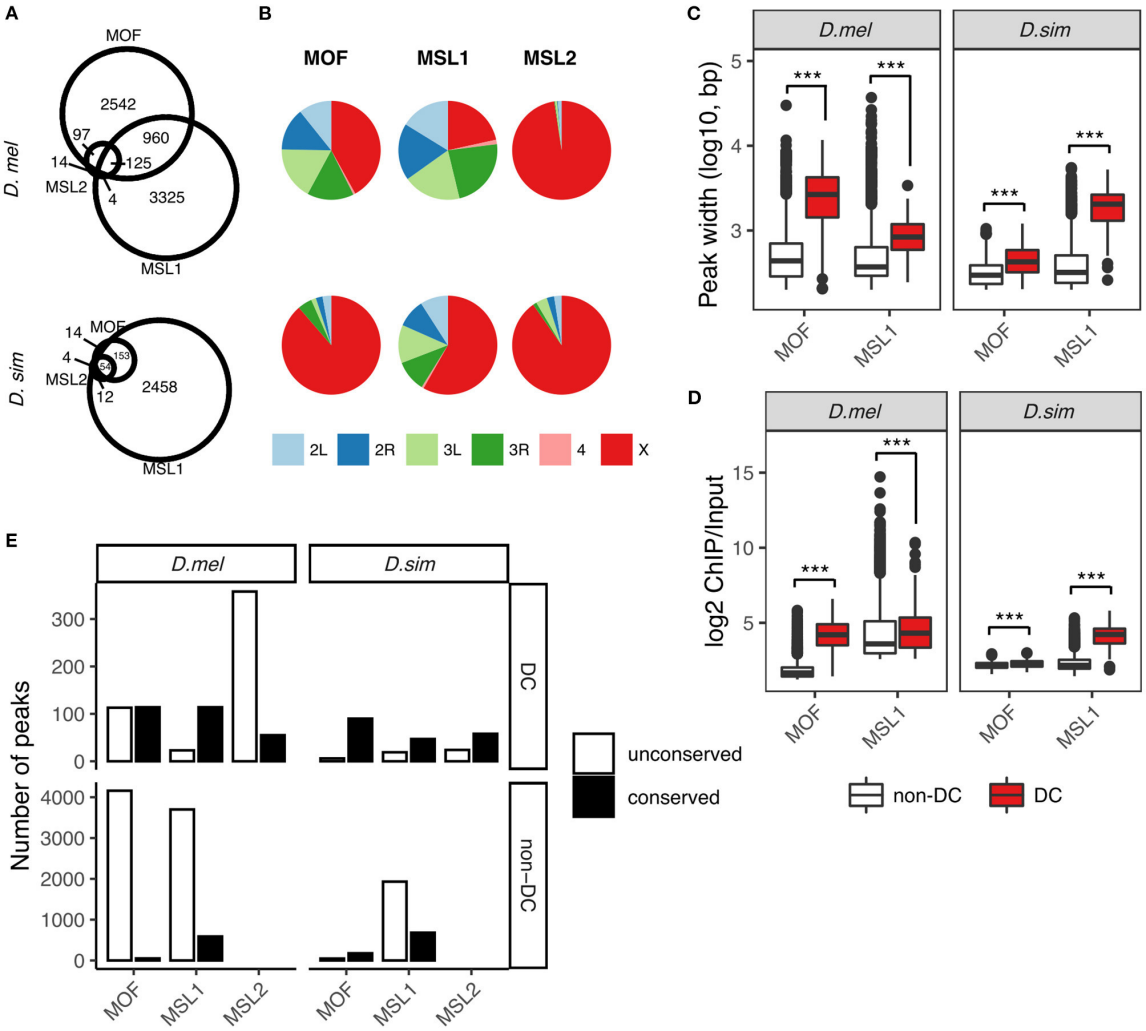
Comparative analysis of MOF, MSL1, and MSL2 binding sites in *D. melanogaster* and *D. simulans***. (A)** Overlaps between MOF, MSL1, and MSL2 binding sites. Numbers reflect merged binding sites (see Materials and Methods). **(B)** Chromosome distribution of MOF, MSL1, and MSL2 binding sites. Boxplot of binding peak widths **(C)** and binding affinities **(D)** of MOF and MSL1 regions involved in dosage compensation (DC, overlapping with MSL2) and unrelated functions (non-DC, non-overlapping with MSL2). Significance was determined by the Mann-Whitney *U*-test: ***P < 0.001. **(E)** Number of conserved DC and non-DC MOF/MSL1 sites in *D. melanogaster* (*D.mel*) and *D. simulans* (*D.sim*).

MSL protein binding sites were considered overlapping if the common regions between two sites were larger than half of the region covered by each site. MOF and MSL1 binding sites were considered DC sites if they overlapped MSL2 bound regions; otherwise, they were considered non-DC sites. Because of the existence of many-to-one or one-to-many relationship when determining overlapping across MSL proteins, individual binding peaks were sorted according to their genomic positions and merged if the common region between adjacent binding peaks was larger than half of the region of either peak. These merged regions are reflected in the binding site numbers presented in Figure 1A.

### Annotations of TE Insertions and Identification of TE-Derived Binding Peaks

We adopted the pipeline described by Kofler et al. (2015) to annotate TE insertions in the *D. melanogaster* (Ensembl BDGP6) and *D. simulans* (FlyBase r2.02) genomes. Briefly, consensus TE sequences (RepBase version 22.02) (Quesneville et al., 2005) were mapped against both reference genomes using RepeatMasker (version open-4.0.7) (Smit et al., 2013–2015). TEs overlapping microsatellites, which were identified by SciRoKo (version 3.4) (Kofler et al., 2007), were identified using bedtools (version 2.25.0) (Quinlan and Hall, 2010) and removed from further analyses. All parameters and filter criteria were the same as described in Kofler et al. (2015). Overlapping TE insertions from the same TE family were merged, and those from different families were resolved by prioritizing the longest TE insertions and iteratively truncating common regions. TE insertions <100 bp were excluded. All TE insertions were classified according to the RepBase (version 22.02) (Quesneville et al., 2005). A binding peak was considered TE-derived if at least one half of the binding region overlapped a TE insertion.

### *De novo* Prediction of Binding Motifs

To measure sequence variation in MSL binding sites, the MOF/MSL1 DC and non-DC sites and MSL2 DC sites in *D. melanogaster* and *D. simulans* were used to *de novo* predict all overrepresented binding motifs using MEME (version 4.12.0, -mod zoops -nmotifs 50 -evt 0.05 -minw 6 -maxw 50 -revcomp) (Bailey et al., 2015). TE-derived binding sequences were excluded from these predictions.

### McDonald-Kreitman (MK) Test

An extended MK test framework (Mackay et al., 2012) was used to detect natural selection of MSL binding sites. We retrieved *D. melanogaster* population genomic data from Lack et al. (197 individuals, DPGP3) (Lack et al., 2015) and *D. simulans* data from Signor et al. (183 individuals, SRP075682) (Signor et al., 2018) (Supplementary Table 1). The *D. melanogaster* lines were from a single ancestral range population from Zambia (Lack et al., 2015) and the *D. simulans* lines were from a North America population (Signor et al., 2018). The raw fastq files were first mapped to the corresponding reference genome (Ensembl BDGP6 for *D. melanogaster* and FlyBase r2.02 for *D. simulans*) by bwa mem (version 0.7.12) (Li, 2013) and processed by samtools (version 1.6) (Li et al., 2009) with default parameters. Reads that were missed by bwa mem were remapped to the corresponding genome again using stampy.py (version 1.0.32) (Lunter and Goodson, 2011). PCR duplicates were removed using Picard MarkDuplicates (version 2.9.0, http://broadinstitute.github.io/picard/). Variant calling of single nucleotide polymorphisms (SNPs) and small insertions and deletions (indels) was performed by GATK (version 3.8) (McKenna et al., 2010) with default parameters. Polymorphism data were extracted from previously published Variant Call Format (VCF) files (McKenna et al., 2010). Singletons and structural variants were discarded. Using *Drosophila yakuba* as the outgroup, divergent sites were inferred from whole genome alignment of *D. yakuba* to *D. melanogaster* and *D. yakuba* to *D. simulans*. Whole genome alignment of *D. yakuba* to *D. melanogaster* was retrieved from https://hgdownload-test.gi.ucsc.edu/goldenPath/dm6/vsDroYak3/. The *D. yakuba* to *D. simulans* alignment was completed independently following the procedure described at http://genomewiki.ucsc.edu/index.php/Whole_genome_alignment_howto. Four-fold degenerate sites in the whole genome of *D. melanogaster* and *D. simulans* were used as the neutral control to make inferences about selection, respectively. Estimates of the fraction of sites that are adaptive fixations (α) were calculated according to Mackay et al. (2012). The MK test (Mackay et al., 2012) was also applied to individual protein-coding genes bearing MOF or MSL non-DC sites in their 5′UTR, CDS, intron, 3′UTR, and 2 kb upstream or 2 kb downstream regions according to annotations of the *D. melanogaster* genome (Ensembl BDGP6). A 2^*^2 contingency table was used to compare polymorphic synonymous and non-synonymous polymorphism, with synonymous and non-synonymous divergence. *P*-values were calculated using a Fisher′s exact test followed by Benjamini-Hochberg correction (Benjamini and Hochberg, 1995) for multiple testing. Only polymorphic sites with available outgroup were used. Software we used to perform site extraction can be found at https://github.com/tonymugen/polyDivExtract.

### Determining Overlap Between *Cis*-Regulatory Elements and Non-DC Sites or TEs

To investigate overlaps between *cis*-regulatory elements and non-DC sites or TEs, three datasets of *cis*-regulatory element annotations in *D. melanogaster* were used (Supplementary Table 1). (i) High quality *cis*-element annotations described by the modENCODE *cis*-regulatory annotation project, including novel promoters, CBP only enhancers, and Class I and II insulators (Negre et al., 2011); (ii) An integrated promoter annotation obtained from Hoskins et al. (2011); (iii) Genome-wide enhancer activity profile of S2 and ovarian somatic cells (OSCs) by STARR-seq (Arnold et al., 2014). Since non-DC sites were determined using dm6 coordinates, the centers/summits of *cis*-elements based on dm3 were translated into dm6 using UCSC liftover tools (https://genome.ucsc.edu/cgi-bin/hgLiftOver). Non-DC sites or TE fragments were considered in common with *cis*-elements if the center/summit of a *cis*-element was located within the region.

### Expression Divergence and Gene Ontology Analysis

Whole female and male adult RNA-seq data from *D. melanogaster* and *D. simulans*, each with two or four biological replicates, were retrieved from the GEO database (GSE28078) (Graveley et al., 2011; Chen et al., 2014) (Supplementary Table 1). Paired reads were mapped *D. melanogaster* (Ensembl BDGP6) and *D. simulans* (FlyBase r2.02) reference genomes using hisat2 (version 2.1.0) (Kim et al., 2015). Expression levels were measured as transcripts per million (TPM) using StringTie (version 1.3.3b, -e -A) (Pertea et al., 2015) and averaged across biological replicates for each species. We classified 11,063 one-to-one orthologous protein-coding genes between *D. melanogaster* and *D. simulans* (Flybase 2017_06, http://flybase.org) into three groups. Transcripts that have a non-DC MOF or MSL1 binding site <2 kb upstream or within the gene body are in a BS+ group, otherwise BS–. If a TE is present within this region, a transcript is marked as TE+. Thus, for example, a transcript with both a non-DC site and a transposon is marked BS+TE+, while one with only a TE is in the BS–TE+ group. Expression divergence between these genes was estimated by 1 – ρ (Spearman's correlation) for each group of genes (Coolon et al., 2014). The distributions of expression divergence per gene group were estimated by drawing 1,000 bootstrap values. To estimate changes in expression levels between *D. melanogaster* and *D. simulans*, TPMs of 11,063 one-to-one orthologous protein-coding genes were normalized to the normal distribution N(0,1) by calculating (x-mean)/sd within species and expression changes were estimated as the differences between these normalized expression values between *D. melanogaster* and *D. simulans*. All statistical analyses were conducted using R (version 3.1.3, https://www.R-project.org). DAVID (version 6.7, http://david.abcc.ncifcrf.gov/) was used to perform a Gene Ontology (GO) enrichment test for genes harboring novel MOF or MSL1 non-DC sites (447 MOF only genes, 3,685 MSL1 only, and 537 genes harboring both). Only the GO terms for “biological processes” were used for the enrichment test.

### Enrichment of Specific TE Families in Non-DC Sites

To estimate whether TE-derived non-DC sites were enriched in specific TE families, a log2 odds-ratio method (Sundaram et al., 2014) was used to identify peak-enriched TE families:

$$\begin{array}{l} LOR_{i,j}\\ =\log_{2}\left(\frac{\begin{array}{c} Number\;of\;non-DC\;sites{\;}^{\prime }i^{\prime }\;derived\;by\;TE\;family{\;}^{\prime }j^{\prime }/\\ Length\;of\;TE\;family{\;}^{\prime }j^{\prime }\;annotated\;in\;genome \end{array}}{\begin{array}{c} Number\;of\;non-DC\;sites\;in\;genome/\\ genome\;size \end{array}}\right) \end{array}$$

We used a threshold of 1.5 for the log2 odds-ratio to identify TE families that are enriched in non-DC sites. Consensus sequences of TE families in *D. melanogaster* were obtained from Repbase (version 22.02) (Quesneville et al., 2005), while those in *D. simulans* were extracted using in-house perl scripts from a sequence alignment generated by MAFFT (version 7.273) (Katoh and Standley, 2013). Scanning TE consensus sequences for occurrence of three frequently detected MSL binding motifs (GAGA, CACA, and GCA) was conducted using FIMO (version 4.12.0, –verbosity 1 –thresh 1.0E-4) (Grant et al., 2011).

### Comparison of TE-Derived Non-DC Binding Sites Between *D. melanogaster* and *D. simulans*

To compare TE-derived non-DC binding sites between *D. melanogaster* and *D. simulans*, the MOF/MSL1 ChIP-seq reads mapping to non-DC binding regions of *D. melanogaster* and *D. simulan*s were extracted by samtools fastq (version 1.6) (Li et al., 2009) and mapped to TE consensus sequences (Repbase version 22.02) (Quesneville et al., 2005) using bowtie (version 1.1.2, -a –best –strata) (Langmead et al., 2009). Non-DC binding peaks in TE consensus sequences were called by macs2 (version 2.1.1.20160309, callpeak -B –nomodel –SPMR -g 684334) (Zhang et al., 2008).

## Results

### Expansion of MSL Complex Binding Sites in *D. melanogaster*

The first step in studying MSL binding site evolution is to identify loci occupied by these proteins in at least two species. We made use of publicly available ChIP-seq data that measure MOF, MSL1, and MSL2 chromatin association in salivary glands from third instar larvae of *D. melanogaster* and *D. simulans* (Figueiredo et al., 2014; Chlamydas et al., 2016). Following the ChIP-seq quality control (QC) guidelines (Landt et al., 2012), cross-correlation analysis revealed that the MSL protein ChIP-seq libraries had QC scores of 0–2 (Supplementary Table 2), indicating that they were of intermediate to very high quality. The normalized ratio between the fragment-length cross-correlation peak and the background cross-correlation (normalized strand coefficient, NSC) and the ratio between the fragment-length peak and the read-length peak (relative strand correlation, RSC) are strong metrics for assessing signal-to-noise ratios in a ChIP-seq experiment (Landt et al., 2012). There was no significant difference for NSC or RSC values between *D. melanogaster* and *D. simulans* (*t*-test, both *P* > 0.05, Supplementary Table 2), suggesting that the overall qualities of the MSL protein ChIP-seq data are comparable between the two species.

We called protein binding sites *de novo* from raw data using macs2 (Zhang et al., 2008) for each species independently (see Materials and Methods). We identified 4,436 MOF, 4,424 MSL1, and 413 MSL2-binding sites in *D. melanogaster*. The number of loci occupied by the MSL complex in *D. simulans* was much lower (MOF: 318, MSL1: 2,677, and MSL2: 82; Figure 1A). It is possible that more MSL binding sites in *D. melanogaster* might be caused by the lower binding affinity in *D. simulans*. To evaluate this, we estimated the gain or loss of bound regions in each species following a previous study on binding site turnover between *Drosophila* species (Bradley et al., 2010) with some modifications (see Materials and Methods). A higher proportion of peaks in *D. melanogaster* (8.2–46.8% depending on the MSL protein) had no ortholog in *D. simulans* than vice versa (6.5–10.1%; Supplementary Table 3). The gain or loss of bound regions for orthologous sequences between *D. melanogaster* and *D. simulans* was rare, with zero to 2.4% peaks found in one species clearly absent or displaced in the other (Supplementary Table 3). The gain/loss rate near genes that flank known HAS was similar to the genome-wide rate, and comparable between species (Supplementary Table 3). These results suggested that binding affinities are sufficiently strong to capture both highly- and poorly-bound regions that are ortholgous between species. Consistent with this, there was no correlation between NSC or RSC values of the ChIP-seq data and the total numbers of detected binding sites of the MSL proteins (Spearman's correlation, both *P* > 0.05, Supplementary Figure 1).

Interestingly, most of the MSL-binding sites specific to *D. melanogaster* are on autosomes (Figure 1B; Supplementary Table 4), suggesting they are unlikely to function in dosage compensation. There is a corresponding drop in the proportions of X-linked MOF- and MSL1-binding sites: 88.7 and 58.2% in *D. simulans* to 42.0 and 21.6% in *D. melanogaster*, respectively (Figure 1B). MSL2 is the only protein of the complex that preferentially targets the X chromosome in both *D. melanogaster* (97.6%) and *D. simulans* (90.2%, Figure 1B), consistent with the idea that it functions almost exclusively in dosage compensation. We then classified MOF and MSL1 binding sites into those involved in dosage compensation (we call them DC sites) and those that likely have additional or independent functions (non-DC sites) according to whether they overlap MSL2-associated loci. We considered two protein binding sites overlapping if at least half of the binding region of one site intersected the other (see Materials and Methods). Such classification may lead to underestimation of the number of non-DC sites, since MSL2 was recently found to target autosomal genes involved in patterning and morphogenesis (Valsecchi et al., 2018); however, the underestimation cannot be large since there are few autosomal MSL2 binding sites (10 in *D. melanogaster* and eight in *D. simulans*, Figure 1B). About 94.0% (3,502 out of 3,724) of MOF binding sites are MSL2-independent in *D. melanogaster*, whereas the fraction (75.6%) is appreciably smaller in *D. simulans* (167 out of 221, Figure 1A). By contrast, the vast majority (97%) of MSL1-associated loci are MSL2-independent in both species (4,285 out of 4,414 in *D. melanogaster*, 2,611 out of 2,677 in *D. simulans*, Figure 1B). The number of binding sites shared by MOF, MSL1, and MSL2, the putative HAS for the MSL-DCC, is two times larger in *D. melanogaster* (125) than in *D. simulans* (54, Figure 1A). In both species, non-DC MOF and MSL1 sites consistently show narrower binding peaks and lower log2 ChIP/Input ratios than DC sites (all Mann-Whitney *U*-test, all *P* < 0.001, Figures 1C,D), suggesting their involvement in distinct molecular functions.

We next sought to investigate how the two classes of binding sites have contributed to the observed expansion in *D. melanogaster*. To do so, we defined the occupancy conservation of MSL binding sites following a previously described procedure (Sundaram et al., 2014) (also see Materials and Methods). Briefly, we considered a binding site conserved if at least half of its binding region in one species overlapped the orthologous region that was also bound by the same MSL protein in the other species. Expansion in either species is then estimated by the number of non-conserved sites. The increase in *D. melanogaster*-specific DC sites is mostly attributable to MSL2-associated regions (*D. melanogaster*: 358 vs. *D. simulans*: 24), followed by MOF (*D. melanogaster*: 113 vs. *D. simulans*: 6) and MSL1 (*D. melanogaster*: 23 vs. *D. sim*: 19; Figure 1E). Much more non-DC sites were found unconserved in either species, particularly in *D. melanogaster* (Figure 1E). We identified 4,159 distinct MOF binding sites in *D. melanogaster* but only 46 in *D. simulans*. The number of non-conserved non-DC MSL1 sites in *D. melanogaster* (3,699) is twice as many as that in *D. simulans* (1,931).

### Rapid Evolution of MSL Binding Sites in *D. melanogaster*

**Figure 2:**
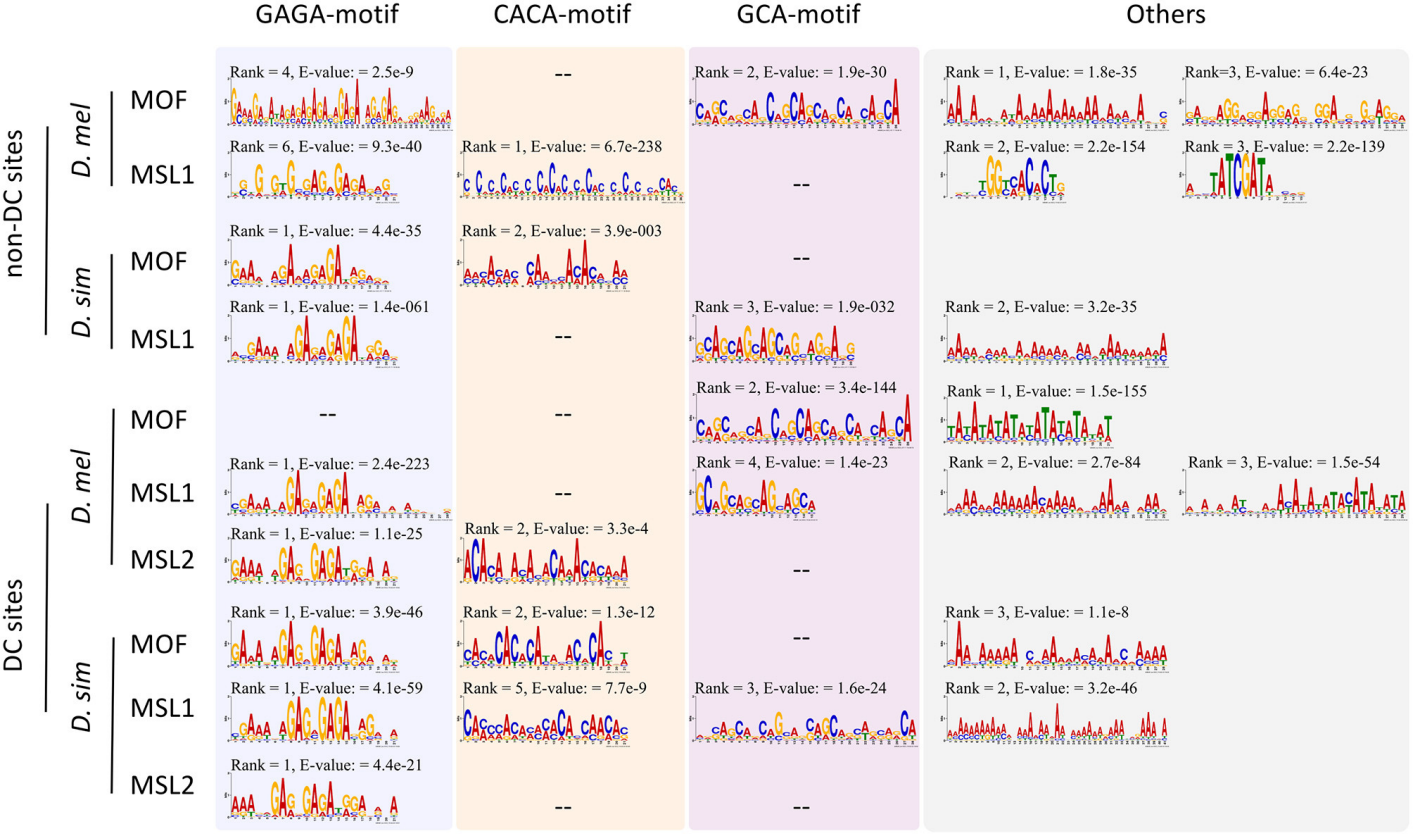
Motifs overrepresented in DC and non-DC sites. Motif ranks are listed according to E-values (<0.05) calculated by MEME (Bailey et al., 2015).

Given the expansion of MSL-binding sites in *D. melanogaster*, we wondered whether DNA sequence motifs targeting the complex to specific regions have diverged (Berg et al., 2004; Tugrul et al., 2015) between *D. melanogaster* and *D. simulans*. Using MEME (Bailey and Elkan, 1994), we identified sequences overrepresented in DC or non-DC sites in *D. melanogaster* and *D. simulans*. As expected, the GAGA motif is enriched in DC sites (Figure 2). This motif is known to associate with high-affinity sites and recruit the MSL complex to chromatin (Alekseyenko et al., 2008; Straub et al., 2008). As for MOF DC sites in *D. melanogaster*, the TATA-box (E-value = 1.5e−155) is more prevalent than GAGA (E-value > 0.05), consistent with the known function of MOF binding to active promoters (Raja et al., 2010; Feller et al., 2012; Straub et al., 2013). In contrast, while the GAGA motif still ranks first at non-DC sites in *D. simulans*, it is not predominant at non-DC loci in *D. melanogaster* (Figure 2). GCA and CACA motifs are overrepresented in these regions instead (Figure 2).

We wondered whether the observed motif turnover can be attributed to positive selection. To estimate the number of adaptive nucleotide substitutions, we applied the extended McDonald-Kreitman (MK) test (Mackay et al., 2012). We used *D. yakuba* as the outgroup and *D. melanogaster* and *D. simulans* population genomic data from Lack et al. (2015) and Signor et al. (2018) to estimate polymorphism levels. The *D. melanogaster* genomes we used were from a single ancestral range population from Zambia (Lack et al., 2015). We used putatively neutrally evolving 4-fold degenerate sites as controls. Because genes on the X and autosomes are subject to distinct evolutionary forces and transcriptional regulation, we paired X-linked silent sites with (almost exclusively X-linked) DC sites and autosomal silent sites with (overwhelmingly autosomal) non-DC regions. Consistent with the expansion of MSL-bound region in *D. melanogaster*, we see signatures of positive selection only at MSL-binding sites specifically in that species (Table 1). Binding sites of all three MSL proteins have a higher D/P ratio than 4-fold degenerate sites (Table 1). Overall, more than 30% (26.4–44.4%) of the observed divergence in MSL binding sites have been fixed adaptively in *D. melanogaster* (Table 1, see Materials and Methods). If we further focus on non-DC sites, the proportion of adaptively fixed substitutions is even higher. Autosomal MOF non-DC sites have a strikingly high proportion (89.9%) of adaptively-fixed divergence, compared to X-linked DC (28.8%) and non-DC sites (27.0%). Similarly, both autosomal and X-linked MSL1 non-DC sites have a higher proportion of adaptively-fixed divergence (46.3 and 40.4%) than DC sites (26.5%) in *D. melanogaster* (Table 1). The D/P ratio of 4-fold sites on the X chromosome (1.754) were not greater than that on autosomes in *D. melanogaster* (1.880; Table 1), which is consistent with previous studies that indicate overall silent nucleotide site diversity on the X in African populations of *D. melanogaster* is similar to, or slightly greater, than that for the autosomes (Campos et al., 2013; Lack et al., 2015; Charlesworth et al., 2018). Only 7.83 (88/1,124) or 3.41% (153/4,493) of protein-coding genes bearing MOF or MSL1 non-DC sites were found to have an excess of non-synonymous divergence compared to the ratio between synonymous and non-synonymous polymorphism (Supplementary Table 5), suggesting that hitchhiking, even if exists, would have limited impact on the non-DC sites. In contrast, both DC and non-DC sites in *D. simulans* exhibit a lower D/P ratio than that of X-linked and/or autosomal 4-fold degenerate sites, suggesting they are under strong purifying selection (Table 1).

**Table 1:** The McDonald Kreitman (MK) test of MOF and MSL1 DC and non-DC sites in *D. melanogaster* (*D. mel*) and *D. simulans* (*D. sim*).

**Species**	**Proteins**	**Chr^**a**^**	**Site type**	**Length**	**D**	**P**	**P_**-singleton**_**	**D/P_**-singleton**_**	**α^**b**^**
*D. mel*	MOF	A+X	4-fold	3,455,307	555,391	374,706	299,171	1.856	
			Total	4,199,054	164,454	80,282	59,102	2.783^***^	0.333
		A	4-fold	2,835,156	458,286	303,736	243,823	1.880	
			Non-DC	2,251,825	23,903	1,520	1,285	18.602^***^	0.899
		X	4-fold	620,151	97,105	70,970	55,348	1.754	
			Non-DC	1,263,250	77,324	43,833	32,152	2.405^***^	0.270
			DC	683,979	63,227	34,929	25,665	2.464^***^	0.288
	MSL1	A+X	4-fold	3,455,307	555,391	374,706	299,171	1.856	
			Total	4,139,270	240,542	91,477	72,545	3.316^***^	0.440
		A	4-fold	2,835,156	458,286	303,736	243,823	1.880	
			Non-DC	3,325,728	187,235	66,297	53,497	3.500^***^	0.463
		X	4-fold	620,151	97,105	70,970	55,348	1.754	
			Non-DC	689,191	41,493	18,484	14,096	2.944^***^	0.404
			DC	124,351	11,814	6,696	4,952	2.386^***^	0.265
	MSL2	X	4-fold	620,151	97,105	70,970	55,348	1.754	
			DC	177,823	17,469	9,191	7,329	2.384^***^	0.264
*D. sim*	MOF	A+X	4-fold	3,396,349	580,321	161,318	115,418	5.028	
			Total	123,565	13,057	5,007	3,119	4.186^***^	−0.201
		A	4-fold	2,812,623	483,206	138,081	101,660	4.753	
			Non-DC	10,110	1,559	532	430	3.626^***^	−0.311
		X	4-fold	583,726	97,115	23,237	13,758	7.059	
			Non-DC	66,219	6,675	2,740	1,623	4.113^***^	−0.716
			DC	47,236	4,823	1,735	1,066	4.524^***^	−0.560
	MSL1	A+X	4-fold	3,396,349	580,321	161,318	115,418	5.028	
			Total	1,433,280	152,204	61,039	39,435	3.860^***^	−0.303
		A	4-fold	2,812,623	483,206	138,081	101,660	4.753	
			Non-DC	323,127	41,098	16,173	12,205	3.367^***^	−0.412
		X	4-fold	583,726	97,115	23,237	13,758	7.059	
			Non-DC	975,417	99,200	40,092	24,221	4.096^***^	−0.724
			DC	134,736	11,906	4,774	3,009	3.957^***^	−0.784
	MSL2	X	4-fold	583,726	97,115	23,237	13,758	7.059	
			DC	22,543	2,442	751	470	5.196^***^	−0.359

*Autosomal binding sites of MSL2 and autosomal DC sites of MOF or MSL1 were not used for the MK test due to their limited number (see Supplementary Table 4)*.

a *The 4th chromosome was excluded from autosomes*.

*** *Fisher's exact test, P < 0.001. To increase statistical power, we used polymorphisms excluding singletons in the MK test (Mackay et al., 2012). 4-fold degenerate sites genome wide, on autosomes (A) and X chromosome (X) were used as neutral control for MK test of the whole genome, autosomal non-DC sites and DC sites and X-linked non-DC sites, respectively*.

b *Fraction of adaptive fixations, calculated using the methods described by Mackay et al. (2012)*.

### *D. melanogaster* Non-DC Sites Overlap Known *Cis*-Regulatory Elements

*D. melanogaster* non-DC sites are enriched for distinct DNA motifs and appear to be under positive selection, suggesting a gain in functionality. Given that MOF and MSL1 have been shown to bind close to active promoters (Straub et al., 2013), we hypothesized that these non-DC regions might play a role in the regulation of gene expression. If that is true, they may act as *cis*-regulatory elements. To test this hypothesis, we looked for overlaps between non-DC sites and the known *cis*-regulatory elements (i.e., promoters, enhancers, and insulators) in *D. melanogaster*. We assembled a list of *cis*-elements by combining sequences identified by modENCODE (Negre et al., 2011), an integrated promoter annotation (Hoskins et al., 2011), and enhancers experimentally verified by STARR-seq (Arnold et al., 2014). We considered an MSL-binding sequence to overlap a *cis*-element if the common region accounted for at least half of the length of the binding site or the *cis*-element. We find that 16.4% (691/4,209) of MOF non-DC sites and 66.9% (2,869/4,287) of MSL1 non-DC loci overlap *cis*-elements (Table 2). MOF-bound regions mostly abut enhancers, while MSL1 non-DC sites are evenly distributed across promoters, enhancers, and insulators (Table 2). This is accordant with the genomic distribution of non-DC sites showing that MOF non-DC sites are mainly located in the intergenic regions (35.42%) or introns (34.55%) while about half of MSL non-DC sites (42.76%) are located within 2 kb upstream of protein coding genes (Supplementary Figure 2). Non-DC sites overlapping *cis*-elements have higher binding affinities than those outside regulatory regions (Mann-Whitney *U*-test, *P* < 0.05, Supplementary Figure 3), suggesting that the overlap is of functional significance.

**Table 2:** Overlap of non-DC MOF/MSL1 binding sites and known *cis*-regulatory elements in *D. melanogaster*.

		**# of Non-DC binding sites**	**Overlaps with** ***cis*****-elements**			
			**Promoter**	**Enhancer**	**Insulator**	**Total^**a**^**
MOF	TE-derived	935	8	172	1	177^*^
	Non-TE-derived	3,274	223	377	45	514
	Sum	4,209	231(33.4%)	549(79.5%)	46(6.7%)	691
MSL1	TE-derived	921	9	196	6	208
	Non-TE-derived	3,366	1,832	1,452	1,773	2,661^***^
	Sum	4,287	1,841(64.2%)	1,648(57.4%)	1,779(62.0%)	2,869

a *Note that one binding site can span more than one cis-element. We counted non-DC sites that overlap multiple cis-elements redundantly and thus the sum of percentages was >100%*.

^*^Fisher's exact test,

* *P < 0.05*;

*** *P < 0.001*.

### Non-DC Sites Associate With Expression Divergence Between *D. melanogaster* and *D. simulans*

**Figure 3:**
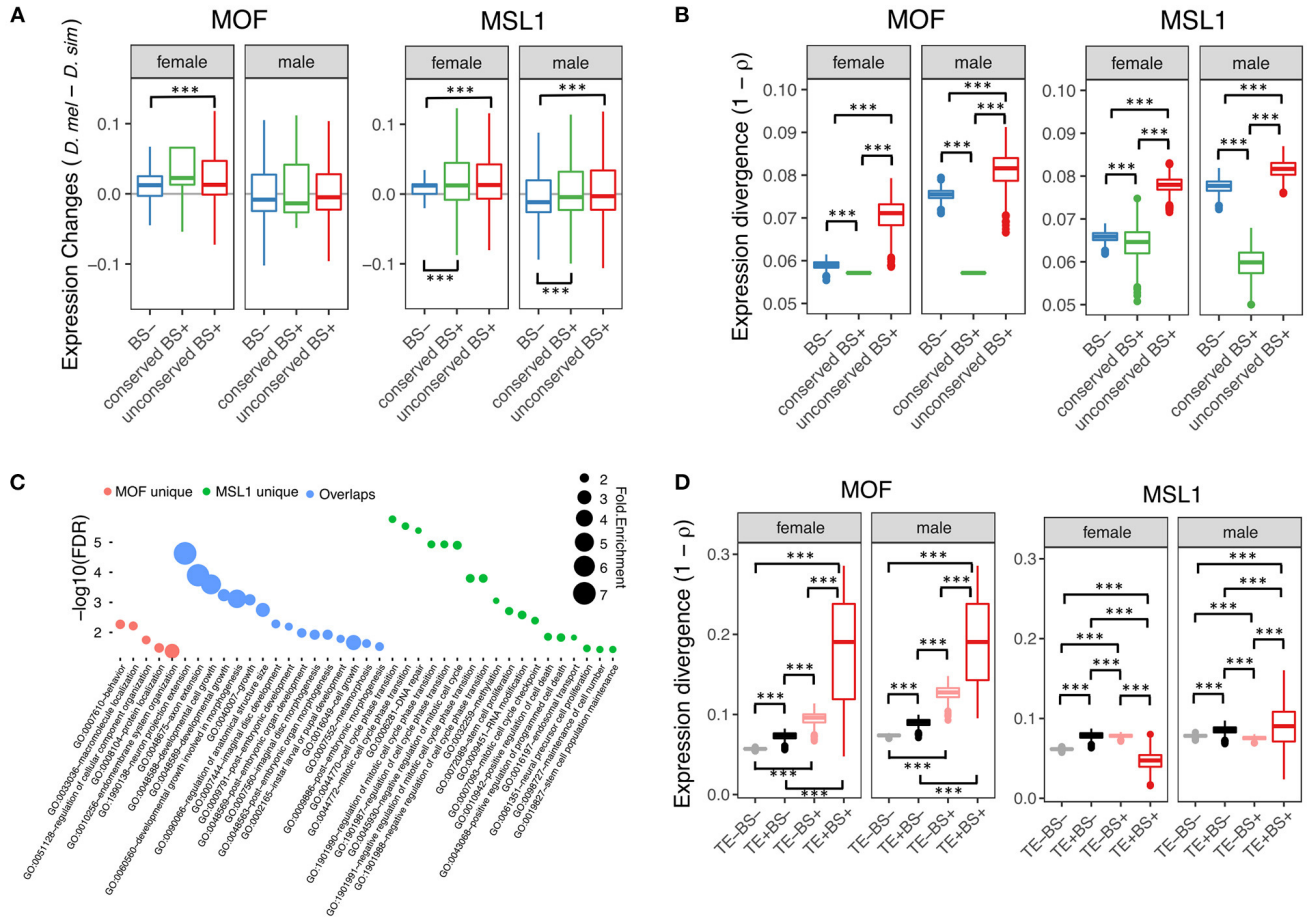
Regulatory role of non-DC sites. **(A)** Changes in gene expression levels between *D. melanogaster* and *D. simulans* in according to presence or absence of non-DC sites. Genes were grouped by presence or absence of non-DC sites 2 kb upstream or in gene bodies of *D. melanogaster*. Expression levels were measured as Transcripts Per Million (TPM) and normalized to a standard normal distribution before between-species comparisons. BS–, without non-DC sites; conserved BS+, with conserved non-DC sites; unconserved BS+, with novel non-DC sites. **(B)** Non-DC sites are associated with increased expression divergence of nearby genes. Gene expression divergence estimated by Spearman's correlation coefficients (1 – ρ) between *D. melanogaster* and *D. simulans*. Genes were grouped as in **(A)**. **(C)** Gene Ontology (GO) analyses of genes with novel non-DC sites. Only significantly enriched GO terms of “biological processes” are shown. MOF unique: genes only harboring MOF non-DC sites; overlaps: genes harboring both MOF/MSL1 non-DC sites; MSL1 unique: genes only harboring MSL1 non-DC sites. **(D)** TE-derived non-DC sites contribute to expression divergence of nearby genes between *D. melanogaster* and *D. simulans*. Expression divergence was calculated as in **(B)** and genes were grouped according to the presence of TE insertions or MOF/MSL1 binding sites close to genes as defined in **(A)**. Significance was determined by the Mann-Whitney *U*-test: ***P < 0.001.

To test whether non-DC site and regulatory element overlap has functional consequences, we examined the effect of autosomal MSL site presence on the *D. melanogaster*/*D. simulans* expression divergence of nearby genes (defined as loci within 2-kb downstream of or containing MSL regions). We obtained published RNA-seq data from whole female and male flies in both species (Graveley et al., 2011; Chen et al., 2014) and estimated gene expression levels as Transcripts Per Million (TPM). We then classified 11,062 protein coding genes one-to-one orthologous between *D. melanogaster* and *D. simulans* according to whether the orthologs in *D. melanogaster* harbored conserved non-DC sites (eight MOF and 689 MSL1 bound genes), non-conserved non-DC sites (984 MOF and 4,222 MSL1) or no MSL sites (9,995 MOF and 5,654 MSL1). Both conserved and non-conserved non-DC MSL1 sites are associated with significantly elevated gene expression levels in male and female *D. melanogaster* (Mann-Whitney *U*-test, both *P* < 0.001, Figure 3A). By contrast, only the non-conserved MOF non-DC sites are associated with higher *D. melanogaster* expression, and that only in females (Mann-Whitney *U*-test, *P* < 0.001, Figure 3A). We also estimated between-species expression divergence of each gene as one minus Spearman's correlation coefficient (1 – ρ) among TPM values as described previously (Coolon et al., 2014). Genes harboring non-conserved non-DC sites exhibit greater expression divergence than loci without non-DC sites (Mann-Whitney *U*-test, *P* < 0.001; Figure 3B), consistent with our expression level observations. The presence of conserved non-DC sites is associated with expression divergence lower than any other category (Mann-Whitney *U*-test, both *P* < 0.001, Figure 3B). These patterns are consistent for MOF and MSL1 in both sexes (Figure 3), suggesting that non-DC sites are indeed involved in regulation of gene expression and its divergence. Gene ontology (GO) analyses revealed that genes harboring non-conserved non-DC sites in *D. melanogaster* are enriched in essential functions (Figure 3C), including “neuron projection extension” (GO:1990138), “axon extension” (GO:0048675) and “developmental cell growth” (GO:0048588) for both MOF and MSL1, “behavior” (GO:0007610) and “macromolecule localization” (GO:0033036) for MOF only, and “RNA processing” (GO:0006396), “mitotic cell cycle” (GO:0000278) and “ribonucleoprotein complex biogenesis” (GO:0022613) for MSL1 only (Figure 3C).

### Non-DC Sites in *D. melanogaster* Overlap Transposons

**Figure 4:**
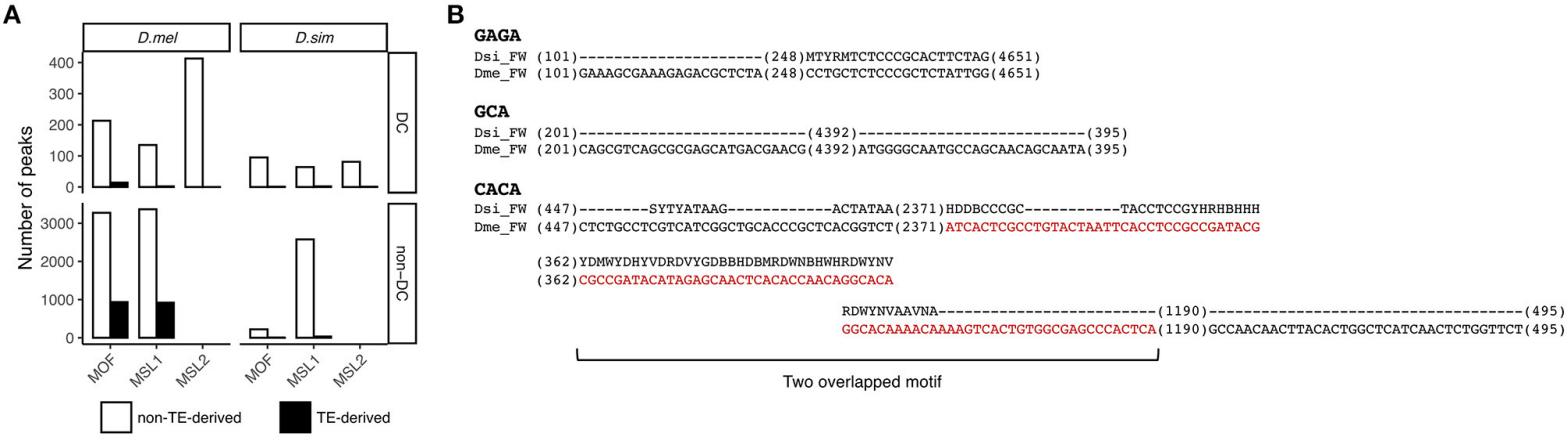
Contribution of TEs to non-DC sites. **(A)** Numbers of DC and non-DC sites located in or outside of TEs in *D. melanogaster* (*D. mel*) and *D. simulans* (*D. sim)*. **(B)** FW transposon family provides MSL binding motifs in *D. melanogaster* but not in *D. simulans*. Sequences alignments show GAGA, GCA, and CACA motifs in the consensus sequences of the FW family in *D. melanogaster* and *D. simulans*. Sequences in red are in MSL1 non-DC binding peaks.

We next wanted to determine the source of the novel autosomal MSL binding sites in *D. melanogaster*. A possible candidate is transposable elements that are known to play a key role in acquisition of novel MSL complex binding sites (Ellison and Bachtrog, 2013, 2019). This hypothesis seems plausible as TE abundance in *D. melanogaster* is twice that in *D. simulans* (Castillo et al., 2011). To test this hypothesis, we annotated 141 and 140 TE families in *D. melanogaster* and *D. simulans*, respectively, using RepeatMasker (Smit et al., 2013–2015) (see Materials and Methods). We define a binding site as residing within a transposon if the center of its read count distribution falls within genomic coordinates of a TE (Sundaram et al., 2014). Most TE-derived MSL-binding sites are MOF and MSL1 associated non-DC regions in *D. melanogaster* (Fisher's exact test, *P* < 0.05 for MOF and *P* < 0.05 for MSL1, Figure 4A).

To determine whether specific TE families are overrepresented at non-DC sites, we calculated an enrichment score estimating the log-odds ratios between the observed number of binding peaks overlapping specific TE families and the number of binding peaks expected by chance (Sundaram et al., 2014), see also Materials and Methods. At a log-odds ratio cutoff of 1.5, representing roughly a 3-fold excess of non-DC binding peaks within a specific TE family over the genome-wide background, we identified 20 TE families enriched for MOF non-DC binding peaks and 17 for MSL1. Most of these families are LTR retrotransposons (Table 3). These transposons have experienced amplification in *D. melanogaster* and more than half of them harbor GAGA, GCA, or CACA motifs that are overrepresented in autosomal MSL complex binding regions as described above (Table 3). For example, we found two GAGA, two GCA, and five CACA motifs in the *FW* non-LTR retrotransposon family consensus sequence in *D. melanogaster*, whereas the homologous regions of *FW* retroelements in *D. simulans* are highly degenerate or divergent (Figure 4B). It appears that TE amplifications have a significant association with the increase of non-DC sites in *D. melanogaster*.

**Table 3:** Summary of motifs and *cis*-regulatory elements specific TE family consensus sequences enriched in non-DC binding sites in *D. melanogaster*.

LOR^*^	**TE family**	**TE class**	**Enriched non-DC BS**	**# of GAGA**	**# of GCA**	**# of CACA**	**# Promoter**	**# Enhancer**	**# Insulator**	**# *cis*-elements**	**TE CopyNumber in *D.mel***	**TE CopyNumber in *D.sim***
7.851	5S_DM	Unclassified	MSL1	0	0	0	0	0	0	0	100	1
6.169	ROO_LTR	LTR	MSL1	0	0	0	4	28	0	31	299	41
5.771	DM297_LTR	LTR	MSL1	0	0	0	0	32	0	32	162	56
5.462	DM412_LTR	LTR	MSL1	0	0	0	0	22	0	22	33	3
5.312	DM412B_LTR	LTR	MSL1	0	0	0	2	23	1	25	48	6
5.192	DMHMR1	Unclassified	MSL1	0	3	0	0	1	0	1	19	6
4.716	PROTOP_A	DNA	MSL1	2	0	0	0	48	0	48	329	87
3.658	FB4_DM	DNA	MSL1	0	0	0	0	2	0	2	212	165
3.155	Jockey	Non-LTR	MSL1	1	2	1	0	7	0	7	128	77
3.026	NTS_DM	Unclassified	MSL1	0	0	0	0	0	0	0	20	0
2.896	PROTOP	DNA	MSL1	3	0	1	0	9	0	9	305	211
2.804	FW	Non-LTR	MSL1	2	2	5	0	68	0	68	232	67
2.354	ROO_I	LTR	MSL1	0	29	1	3	27	0	30	240	49
2.279	DOC	Non-LTR	MSL1	1	1	0	0	10	0	10	165	27
2.200	MDG1_LTR	LTR	MSL1	0	0	0	0	0	0	0	88	12
2.057	S_DM	DNA	MSL1	1	0	0	0	0	0	0	144	19
1.695	STALKER4_LTR	LTR	MSL1	0	1	0	0	2	0	2	172	10
7.848	5S_DM	Unclassified	MOF	0	0	0	0	0	0	0	100	1
6.186	ROO_LTR	LTR	MOF	0	0	0	3	18	0	20	299	41
5.967	NOMAD_LTR	LTR	MOF	0	0	1	0	8	0	8	61	4
5.935	DM412_LTR	LTR	MOF	0	0	0	0	28	0	28	33	3
5.812	DM297_LTR	LTR	MOF	0	0	0	0	22	0	22	162	56
5.603	COPIA_DM_LTR	LTR	MOF	0	1	0	0	0	0	0	76	4
5.576	DM412B_LTR	LTR	MOF	0	0	0	0	27	0	27	48	6
5.516	COPIA2LTR_DM	LTR	MOF	1	0	0	0	0	0	0	45	14
5.459	MDG1_LTR	LTR	MOF	0	0	0	0	1	0	1	88	12
4.994	LTRMDG3_DM	LTR	MOF	0	0	0	0	0	0	0	37	8
4.618	QUASIMODO_LTR	LTR	MOF	0	0	13	0	32	0	32	127	8
3.982	INVADER1_LTR	LTR	MOF	0	0	0	0	0	0	0	115	34
3.133	HETRP_DM	Unclassified	MOF	10	3	1	0	1	0	1	28	7
3.053	NTS_DM	Unclassified	MOF	0	0	0	0	0	0	0	20	0
2.455	Jockey	Non-LTR	MOF	1	2	1	0	0	0	0	128	77
2.325	FB4_DM	DNA	MOF	0	0	0	0	0	0	0	212	165
2.245	FW	Non-LTR	MOF	2	2	5	0	15	0	15	232	67
2.091	ROO_I	LTR	MOF	0	29	1	1	8	0	9	240	49
1.838	PROTOP_A	DNA	MOF	2	0	0	0	5	0	5	329	87
1.754	TART_DV	Non-LTR	MOF	2	423	4	5	10	1	13	799	747

* *log2 Odds-ratio, defined as the ratio between (# of TE-derived BS/total TE length) and (# of non-DC sites/genome size)*.

MOF TE-derived non-DC sites are slightly more likely than non-TE associated regions to overlap *cis*-elements (Fisher's exact test, *P* < 0.05, Table 2), while transposon MSL1 loci are significantly less likely to do so (Fisher's exact test, *P* < 0.001, Table 2). We further looked for overlap between specific TE families enriched for non-DC sites and known *cis*-elements in *D. melanogaster*. The vast majority of these TE families not only overlaps enhancers but also harbors MSL-binding motifs in their consensus sequences (Table 3). We wondered whether TE-derived non-DC sites are associated with larger gene regulation effects than non-TE-derived non-DC sites. Genes with TE-derived non-DC MOF binding sites show significantly higher expression divergence between *D. melanogaster* and *D. simulans* than genes with non-TE-derived or no MOF binding sites in both female and male flies (Mann-Whitney *U*-test, all *P* < 0.005, Figure 3D). However, no consistent pattern was found for MSL1 non-DC sites (Figure 3D), probably because MSL1 only has a small number of TE-derived non-DC sites (Table 3) and exhibits an even distribution of *cis*-regulatory element types (Table 2). Taken together, our results strongly suggest that transposable elements are important drivers of non-DC site expansion in *D. melanogaster* and this expansion in turn affects gene expression divergence from *D. simulans*.

## Discussion

Pleiotropic effects are observed if a gene product participates in multiple biological processes. This can be achieved, for example, if a protein engages in a variety of protein-protein or protein-DNA interactions (He and Zhang, 2006). DNA binding sites of a protein can thus illuminate the impact of pleiotropy on molecular evolution. We studied evolution of dosage compensation complex binding sites in *D. melanogaster* and *D. simulans*. We found dramatic expansion of MSL complex binding sites on the *D. melanogaster* branch relative to *D. simulans*. This expansion is dominated by almost exclusively autosomal sites that have likely acquired a novel function unrelated to dosage compensation (non-DC sites). These sites appear to be under strong positive selection, overlap *cis*-regulatory elements and transposons, and associate with divergent gene expression. The rapid binding site evolution we uncovered mirrors adaptive evolution of the proteins themselves (Levine et al., 2007; Rodriguez et al., 2007) and suggests that MSL complex proteins in *D. melanogaster* are under selection for a novel function. Concerted rapid evolution of regulatory sites and *trans*-factors appears to be a recurrent theme in dosage compensation, since adaptive evolution of *roX* genes and their putative binding region has been reported in *D. melanogaster* (Levine et al., 2007), along with rapid turnover of *roX* binding sites in diverse *Drosophilid* species (Quinn et al., 2016).

One caveat of this study is that the ChIP-seq data we used are from two studies: the MSL1 data in *D. melanogaster* are from Chlamydas et al. (2016) while all the other data are from Figueiredo et al. (2014), and have no replicate. Therefore, the observed expansion of MSL binding sites in *D. melanogaster* could be resulted from antibody-specific and/or lab-specific batch effects. Nevertheless, the gain or loss of orthologous bound regions are fewer than 3% between genomes (Supplementary Table 3), suggesting that the overestimation of MSL binding sites in *D. melanogaster* should be limited. No correlation between NSC/RSC metrics and the number of X-linked or all MSL binding sites across species (Spearman's correlation, all *P* > 0.05; Supplementary Figure 1) also supports that experimental artifacts contribute little to our estimation. Moreover, the fraction (41.8%) of autosomal binding sites of MSL1 was substantial in *D. simulans* (Figure 1; Supplementary Table 4), indicating that the existence of MSL binding sites involved in non-DC function is common in both *Drosophila* species. Future study that directly compares the MSL binding sites between *D. melanogaster* and *D. simulans* using species-specific antibodies with replicates needs to be done to validate the divergent evolutionary pattern of DC and non-DC sites between species. Our results, although need to be considered with cautions, may provide insights about potential features that drive rapid turnover of MSL complex binding sites.

While both DC and non-DC sites appear to be evolving adaptively, selection strength is not the same across these groups of sites. Looking at patterns of excess divergence across site categories offers a way to reveal these discrepancies. Proportions of adaptively fixed sites are very similar (between 26 and 29%) among DC loci bound by MSL1, MOF, and MSL2. This is not surprising since dosage compensation requires all three genes, although overlap between their binding sites is not complete. Thus, there is evidence for significant concerted adaptive evolution of MSL complex proteins and their DC sites. In contrast, MOF and MSL1 non-DC sites show, to different extents, more dramatic signatures of positive selection. Almost all (90%) autosomal MOF non-DC sites have been fixed adaptively and the proportion is also high (46%) for MSL1 non-DC sites. This suggests that some dosage compensation unrelated functions of these proteins are under even more intense positive selection in *D. melanogaster* than the selection driven by their participation in dosage compensation. Moreover, these functions need not be common between MSL1 and MOF. This is consistent with the observation that only about a quarter of autosomal MSL1 and MOF sites overlap each other (960 common sites out 3,502 MOF and 4,285 MSL1 loci). Both proteins bind non-DC sites enriched for DNA sequences different from the GAGA motif found at DC sites and both are found at autosomal promoters (Straub et al., 2013). The extensive overlap between non-DC sites and *cis*-regulatory elements, together with our observation that non-DC sites associate with expression divergence, suggest a potential role of autosomal binding by MSL complex proteins in genome wide transcriptional regulation. Taken together, these data indicate that evolution of the MSL complex proteins is dominated by positive selection for multiple functions. It should be noted that the *D. melanogaster* lines used for MK test were from a single ancestral range population from Zambia (Lack et al., 2015) and thus demography should have little effect, if any, on the estimation of positive selection in *D. melanogaster*. In contrast, the *D. simulans* lines were from a North America population that was suggested to have undergone a recent population bottleneck, along with an excess intermediate frequency polymorphisms (Signor et al., 2018), which may comfound the detection of positive selection.

The most dramatic signatures of positive selection are localized to the N-terminal domains of MSL1 and MSL2. These domains are essential for these proteins' interaction with each other and their targeting to the X chromosome (Rodriguez et al., 2007). Our results suggest that MSL1 autosomal non-DC sites evolve faster than the X-linked DC sites, suggesting that selection on its protein sequence may not be driven by dosage compensation related activity. This idea is consistent with the finding that the direct interaction of MSL2 with HAS on the X chromosome is coincident with MLE rather than MSL1 binding (Straub et al., 2013). It is intriguing in this context that some autosomal MSL2 sites do exist and are deeply conserved between *Drosophila* and mouse (mESCs, mouse embryonic stem cells) (Valsecchi et al., 2018). Disentangling the conserved and evolutionarily labile MSL complex functions is thus an interesting challenge for future research.

The autosomal binding and function of MOF appear to be independent of MSL1. Indeed, there is no evidence for positive selection on amino acids at the MSL1-MOF interaction surface (Rodriguez et al., 2007). Remarkably, autosomal MOF non-DC sites exhibit the most dramatic signatures of positive selection (90% sites were fixed adaptively) whereas the proportion of adaptively fixed divergence for X-linked MOF non-DC sites (27%) is comparable with that for DC sites (26–29%). No such difference was found for MSL1 non-DC sites between autosomes (46%) and the X chromosome (40%). The unusually strong selection on autosomal binding of MOF may be associated with its participation in the non-specific lethal (NSL) complex, a transcription co-activator dedicated to particular housekeeping genes (Feller et al., 2012). This view is supported by the observation that MOF non-DC sites predominantly overlap with enhancers (Table 2). Thus, MOF may be an example of a pleiotropic protein that interacts with several partners and is simutaneoulsy subject to distinct selective pressures. The MOF-containing NSL complex may poss cell-type specific and cellular context-specific functions (Sheikh et al., 2019). Future study on potential adaptive evolution of MOF-NSL acetylation targets as well as NSL binding partners in *D. melanogaster* will provide a better understanding of the NSL complex functions.

The selective pressures that drive adaptive evolution of MSL proteins and their binding sites in *D. melanogaster* remain elusive. Genetic conflict with TEs is a possible force driving this adaptive evolution (Rodriguez et al., 2007; Bachtrog, 2008). Consistent with this notion, we found that TEs have contributed to a significant fraction of non-DC sites, 22.2% for MOF and 21.5% for MSL1, but not to DC sites (Figure 4A). This differs from the pattern in *D. miranda*, where the co-option of a mutant helitron TE has facilitated the acquisition of MSL binding sites on the neo-X chromosome (Ellison and Bachtrog, 2013). TE-mediated regulatory rewiring also contributes to the dosage-compensation network on the neo-X of *D. robusta* (Ellison and Bachtrog, 2019).

While molecular mechanisms and consequences of MSL protein action at their autosomal loci remain unclear, early embryogenesis, where functions associated with autosomal binding of MSL proteins can work together with dosage compensation to maintain fitness, is a possible candidate. Autosomal binding of MOF in both sexes can dampen transcriptional noise (Lee et al., 2018). This may aid in canalization of segmentation (Lott et al., 2007; Manu et al., 2009) which is necessary because embryo size varies across *Drosophila* species (Markow et al., 2009). Thus, MOF-associated complexes may play a vital role in early embryogenesis by fine-tuning gene expression. Indeed, NSL mostly acts on housekeeping genes in pluripotent mESCs while MSL preferentially binds to mESC-specific and bivalent genes (Ravens et al., 2014). Furthermore, MSL2 regulates autosomal genes mainly involved in patterning and morphogenesis and this regulation is remarkably similar in *Drosophila* and mESCs (Valsecchi et al., 2018). In addition to transcription regulation by autosomal binding, dosage compensation via MOF-mediated H4K16 acetylation is required for males to develop into adults during the onset of zygotic gene transcription in *Drosophila* (Copur et al., 2018). Regardless of the exact mechanism, it is clear that MSL proteins affect multiple traits. The distinct yet coherent patterns of adaptive evolution of the DC and non-DC sites suggest that pleiotropy does not necessarily constrain protein evolution. Rather, synergistic pleiotropy can promote rapid protein sequence evolution as it does in phenotypic evolution. One such case is an *Eda* haplotype under strong selection in stickleback fishes with large effects on three phenotypic traits (Archambeault et al., 2020). An in-depth examination of such examples is an important avenue of further research and will enhance our understanding of the interplay between pleiotropy and adaptation.

## Data Availability Statement

The original contributions presented in the study are included in the article/Supplementary Material, further inquiries can be directed to the corresponding author.

## Author Contributions

AD, YW, AG, ZL, and TT conceived and designed the study. AD and AG developed the methodology. AD, YW, AG, and TT analyzed and interpreted the data. AD, AG, and TT wrote, reviewed, and revised the manuscript. All authors contributed to the article and approved the submitted version.

## Conflict of Interest

The authors declare that the research was conducted in the absence of any commercial or financial relationships that could be construed as a potential conflict of interest.
