# Supplementary material for: Rapid Evolution of Autosomal Binding Sites of the Dosage Compensation Complex in Drosophila melanogaster and Its Association With Transcription Divergence
Source: Front Genet. 2021 Jun 14;12:675027. doi: 10.3389/fgene.2021.675027 (PMC8238462; doi:10.3389/fgene.2021.675027)
Supplement: Supplementary file 1 [file Data_Sheet_1.zip › Suppl. Tables & Figures.DOCX]

**Supplemental Information**

**Supplemental Figures
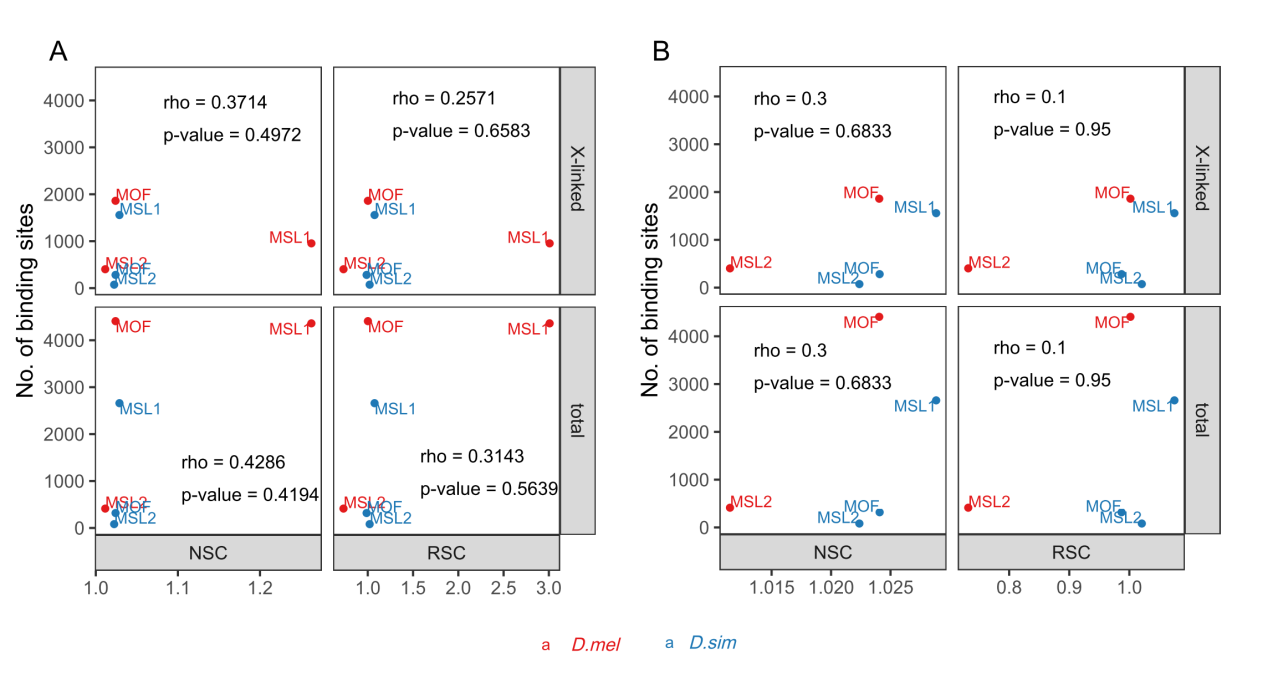
**

**Figure S1.** Spearman’s rank correlation analysis between the number of X-linked or total binding sites and ChIP-seq data quality metrics measured by cross-correlation analysis of SPP (version -1.15.2) (Kharchenko et al., 2008). Spearman rank correlations (rho) and p-values were shown in figures. Points labeled in red are from *D. melanogaster* (*D. mel*) while those in blue are from *D. simulans* (*D. sim*). NSC, normalized strand coefficient; RSC, relative strand correlation.

**
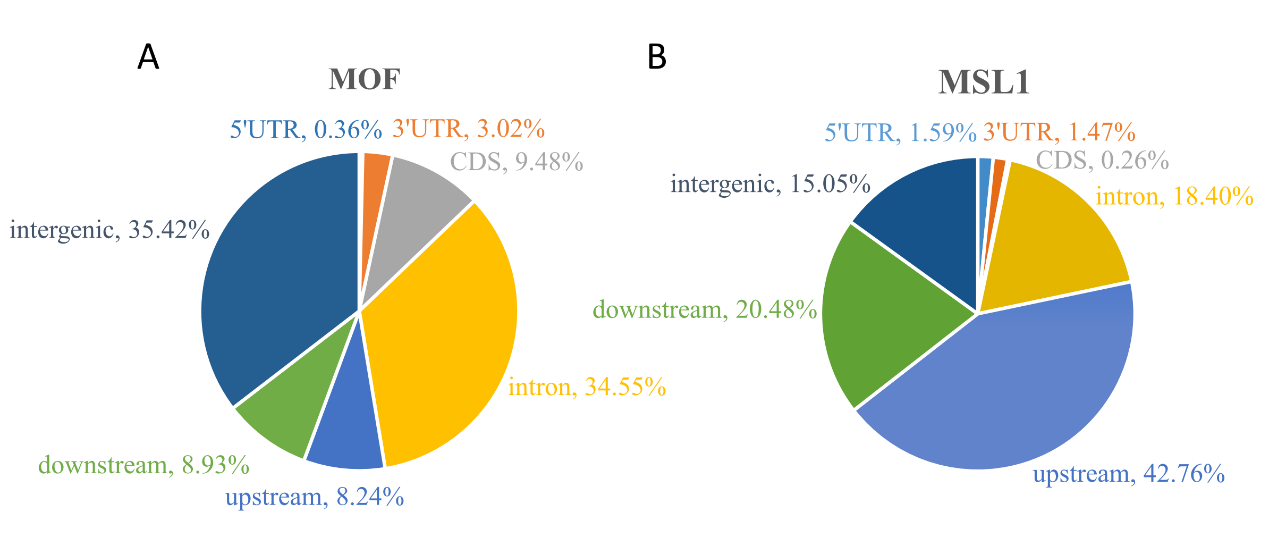
**

**Figure S2.** The genomic distribution of MOF (A) and MSL1 (B) non-DC sites. Upstream: 2kb upstream of a protein-coding gene; downstream: 2kb downstream of a protein-coding gene; intergenic: genomic regions located between protein-coding genes, excluding 2kb upstream and 2kb downstream regions. CDS, coding sequences; UTR, untranslated region.


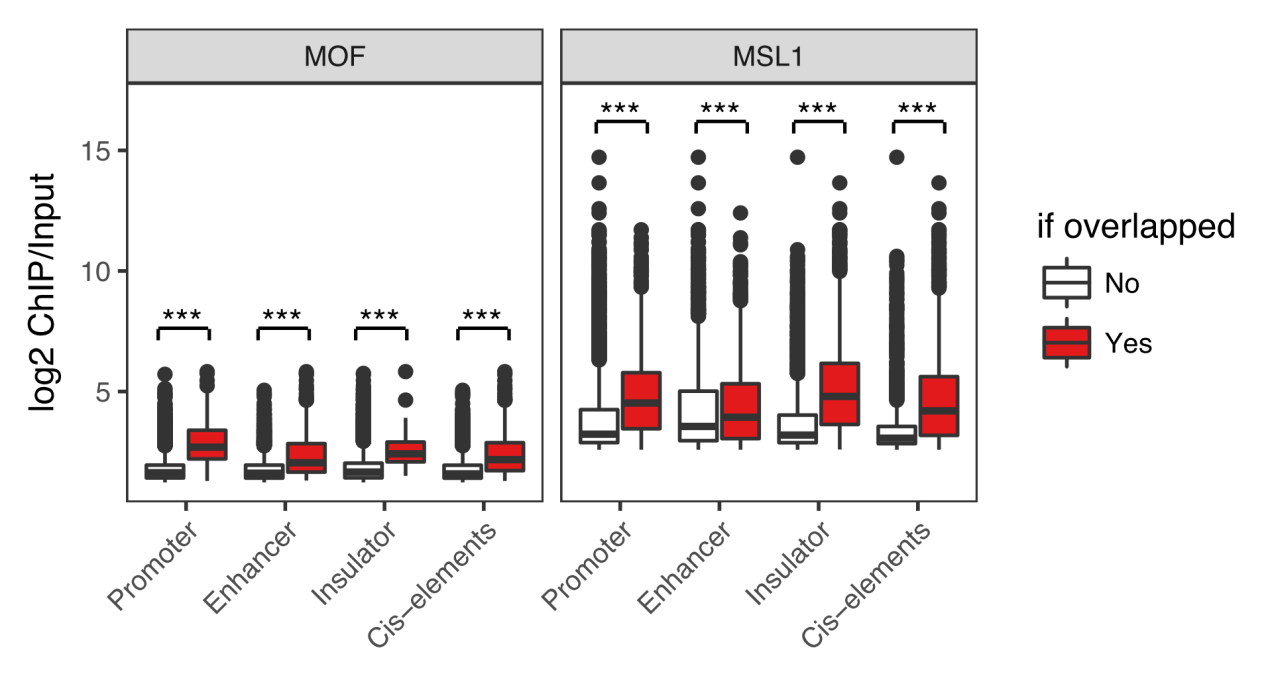


**Figure S3.** Boxplot of binding affinities of MOF (left) and MSL1 (right) non-DC sites that overlap cis-elements. A Mann-Whitney *U* test was used. Asterisks indicate *P*-values (***, *P* < 0.001).

**Supplemental Tables**

**Table S1.** Data summary.

| Data type | Description | Reference |
| --- | --- | --- |
| ChIP-seq | Used to identify binding sites of MOF, MSL1 and MSL2 for *D. melanogaster* and *D. simulans* | (Figueiredo et al., 2014; Chlamydas et al., 2016) |
| WGS | *D. melanogaster* populations from DPGP3 | (Lack et al., 2015) |
| WGS | *D. simulans* populations from Zuma organic orchard in Zuma beach, California | (Signor et al., 2018) |
| ChIP-seq/ChIP-chip | Novel promoters, CBP only enhancers, and Class I and II insulators as showed in supplementary table 8, 10, 11 and 13. | (Negre et al., 2011) |
| CAGE/ RLM-RACE/ ESTs | Integrated promoter annotation as showed in supplementary data file 3. | (Hoskins et al., 2011) |
| STARR-seq | Genome-wide enhancer activity profile of S2 and ovarian somatic cells (OSCs) as listed in supplementary data set 1. | (Arnold et al., 2014) |
| RNA-seq | Used to call expression of female and male adults in *D. melanogaster* | (Graveley et al., 2011; Chen et al., 2014) |
| RNA-seq | Used to call expression of female and male adults in *D. simulans* | (Graveley et al., 2011; Chen et al., 2014) |

**Table S2.** Quality control metrics of the ChIP-seq data used in this study. Cross-corelation analysis as impleted in SPP (Kharchenko et al., 2008) was used to asses read clustering in ChIP-seq data (Landt et al., 2012). Quality tag values of 0, 1 and 2 indicate a medium, high and very high degree of read clustering, respectively. NSC, normalized strand coefficient; RSC, relative strand correlation; *D. mel, Drosophila melanogaster; D. sim, D. simulans.*

| Data Source | Proteins | NSC | | RSC | | QualityTag | |
| --- | --- | --- | --- | --- | --- | --- | --- |
|  |  | *D. mel* | *D. sim* | *D. mel* | *D. sim* | *D. mel* | *D. sim* |
| Figueiredo *et al.* (2014) | input | 1.010 | 1.022 | 0.685 | 1.023 | 0 | 1 |
|  | MOF | 1.024 | 1.024 | 1.002 | 0.987 | 1 | 0 |
|  | MSL1 |  | 1.029 |  | 1.075 |  | 1 |
|  | MSL2 | 1.012 | 1.022 | 0.732 | 1.021 | 0 | 1 |
| Chlamydas *et al.* (2016) | input | 1.018 |  | 2.271 |  | 2 |  |
|  | MSL1 | 1.263 |  | 3.009 |  | 2 |  |

**Table S3.** Gain and loss of peaks of MSL proteins.

| Protein | Species | Total peaks called by MACS2 | Absent in otholog (HAS gene) | Absent in otholog (others) | Without otholog |
| --- | --- | --- | --- | --- | --- |
| MOF | *D. mel* | 4436 | 1/422 (0.2%) | 47/1935 (2.4%) | 2079 (46.8%) |
|  | *D. sim* | 318 | 0/153 (0.0%) | 1/131 (0.7%) | 34 (10.1%) |
| MSL1 | *D. mel* | 4424 | 0/199 (0.0%) | 37/3294 (1.1%) | 931 (21.0%) |
|  | *D. sim* | 2677 | 3/444 (0.7%) | 2/2060 (0.1%) | 173 (6.5%) |
| MSL2 | *D. mel* | 413 | 0/188 (0.0%) | 0/191 (0.0%) | 34 (8.2%) |
|  | *D. sim* | 82 | 0/43 (0.0%) | 0/31 (0.0%) | 8 (9.8%) |

HAS gene: genes located within 3kb upstream/downstream of HAS

Peaks were called as absent if the binding signal was reduced 10-fold or more in its ortholog. The denominators include only peaks where orthologs could be identified (Bradley et al., 2010).

**Table S4**. Chromosome distribution of DC and non-DC sites (peaks) of MSL proteins in *D. melanogaster* (*D.mel*) and *D. simulans* (*D.sim*). The numbers of MSL1 and MOF non-DC sites overlapping each other are given in parentheses.

| Site type | Protein | *D.mel* | | |  | *D.sim* | | |
| --- | --- | --- | --- | --- | --- | --- | --- | --- |
|  |  | A | X | total |  | A | X | total |
| DC sites | MOF | 1 | 226 | 227 |  | 0 | 96 | 96 |
|  | MSL1 | 0 | 137 | 137 |  | 4 | 62 | 66 |
|  | MSL2 | 10 | 403 | 413 |  | 8 | 74 | 82 |
| Non-DC sites | MOF | 2574 (1329) | 1635(338) | 4209(1667) |  | 36(24) | 186(184) | 222(208) |
|  | MSL1 | 3470(723) | 817(239) | 4287(962) |  | 1115(24) | 1496(129) | 2611(153) |

**Table S5.** McDonald Kreitman (MK) test of genes harboring MOF/MSL1 non-DC sites.

**Supplemental References**

Arnold, C.D., Gerlach, D., Spies, D., Matts, J.A., Sytnikova, Y.A., Pagani, M., et al. (2014). Quantitative genome-wide enhancer activity maps for five *Drosophila* species show functional enhancer conservation and turnover during cis-regulatory evolution. Nat Genet. 46, 685-692. doi: 10.1038/ng.3009.

Bradley, R.K., Li, X.Y., Trapnell, C., Davidson, S., Pachter, L., Chu, H.C., et al. (2010). Binding site turnover produces pervasive quantitative changes in transcription factor binding between closely related *Drosophila* species. PLoS Biol. 8, e1000343. doi: 10.1371/journal.pbio.1000343.

Chen, Z.X., Sturgill, D., Qu, J., Jiang, H., Park, S., Boley, N., et al. (2014). Comparative validation of the D. melanogaster modENCODE transcriptome annotation. Genome Res. 24, 1209-1223. doi: 10.1101/gr.159384.113.

Chlamydas, S., Holz, H., Samata, M., Chelmicki, T., Georgiev, P., Pelechano, V., et al. (2016). Functional interplay between MSL1 and CDK7 controls RNA polymerase II Ser5 phosphorylation. Nat Struct Mol Biol. 23, 580-589. doi: 10.1038/nsmb.3233.

Figueiredo, M.L., Kim, M., Philip, P., Allgardsson, A., Stenberg, P., and Larsson, J. (2014). Non-coding roX RNAs prevent the binding of the MSL-complex to heterochromatic regions. PLoS Genet. 10, e1004865. doi: 10.1371/journal.pgen.1004865.

Graveley, B.R., Brooks, A.N., Carlson, J.W., Duff, M.O., Landolin, J.M., Yang, L., et al. (2011). The developmental transcriptome of *Drosophila melanogaster*. Nature. 471, 473-479. doi: 10.1038/nature09715.

Hoskins, R.A., Landolin, J.M., Brown, J.B., Sandler, J.E., Takahashi, H., Lassmann, T., et al. (2011). Genome-wide analysis of promoter architecture in *Drosophila melanogaster*. Genome Res. 21, 182-192. doi: 10.1101/gr.112466.110.

Kharchenko, P.V., Tolstorukov, M.Y., and Park, P.J. (2008). Design and analysis of ChIP-seq experiments for DNA-binding proteins. Nat Biotechnol. 26, 1351-1359. doi: 10.1038/nbt.1508.

Lack, J.B., Cardeno, C.M., Crepeau, M.W., Taylor, W., Corbett-Detig, R.B., Stevens, K.A., et al. (2015). The *Drosophila* genome nexus: a population genomic resource of 623 *Drosophila melanogaster* genomes, including 197 from a single ancestral range population. Genetics. 199, 1229-1241. doi: 10.1534/genetics.115.174664.

Landt, S.G., Marinov, G.K., Kundaje, A., Kheradpour, P., Pauli, F., Batzoglou, S., et al. (2012). ChIP-seq guidelines and practices of the ENCODE and modENCODE consortia. Genome Res. 22, 1813-1831. doi: 10.1101/gr.136184.111.

Negre, N., Brown, C.D., Ma, L., Bristow, C.A., Miller, S.W., Wagner, U., et al. (2011). A cis-regulatory map of the *Drosophila* genome. Nature. 471, 527-531. doi: 10.1038/nature09990.

Signor, S.A., New, F.N., and Nuzhdin, S. (2018). A Large Panel of *Drosophila simulans* Reveals an Abundance of Common Variants. Genome Biol Evol. 10, 189-206. doi: 10.1093/gbe/evx262.
